# P05.51. Eligibility assessment for clinical trials involving chronic pain at a chiropractic research center: a consensus case review process

**DOI:** 10.1186/1472-6882-12-S1-P411

**Published:** 2012-06-12

**Authors:** R Vining, K Pohlman, S Salsbury, E Potocki, M Seidman, A Morgenthal, M Hondras

**Affiliations:** 1 Palmer Center for Chiropractic Research, Davenport, USA

## Purpose

To describe a consensus-based case review development process that applies eligibility and diagnostic criteria for diverse clinical trials of chiropractic care for chronic pain conditions.

## Methods

A multidisciplinary team of investigators and clinical research staff provide iterative feedback to develop case review processes for application across complex clinical trials involving biomechanical testing and/or spinal manipulation. Investigative team members design eligibility criteria and operational definitions consistent with the specific aims of the trial. These parameters are codified in study protocols and programmed into secure, web-based data collection systems that record eligibility decisions. The clinical team develops study-specific procedures, documentation templates, and flow-charts for conducting meetings. Research records are uploaded onto a web server for panel members to view during the meeting.

## Results

Our case review panel consists of research clinicians, project managers, study coordinators and the senior clinician who convene twice weekly to discuss participants who remain eligible following completion of baseline visits. The research clinician who performed the eligibility examination begins case review with an oral presentation of the case and leads the case review panel through a discussion of safety and compliance issues, eligibility concerns, diagnosis determination, and clinical precautions. Panel members provide clinical recommendations and determine final eligibility status by consensus vote. The senior clinician determines eligibility only in cases where consensus (80%) is not reached. Through this process, our research clinic presented 534 cases, excluded 174 participants, and allocated 291 into two phase II clinical trials and six feasibility studies over a three year period.

## Conclusion

Our case review process utilizes the combined scientific and clinical experience of panel members for consistent eligibility determination, thereby reducing selection bias and ensuring that participants are appropriate candidates for study procedures. The consensus building process creates a collegial environment that enhances clinical trial management and fosters rigorous clinical research.

